# Locomotor-cognitive dual-tasking in children with developmental coordination disorder

**DOI:** 10.3389/fpsyg.2024.1279427

**Published:** 2024-03-06

**Authors:** Emily Subara-Zukic, Thomas B. McGuckian, Michael H. Cole, Bert Steenbergen, Peter Henry Wilson

**Affiliations:** ^1^Healthy Brain and Mind Research Center, School of Behavioral and Health Sciences, Australian Catholic University, Melbourne, VIC, Australia; ^2^Behavioral Science Institute, Radboud University, Nijmegen, Netherlands

**Keywords:** developmental coordination disorder, dual-tasking, motor control, locomotion, cognitive control, augmented-reality

## Abstract

**Introduction:**

Children with Developmental Coordination Disorder (DCD) demonstrate deficits in predictive motor control and aspects of cognitive control compared with their typically developing (TD) peers. Adjustment to dynamic environments depends on both aspects of control and the deficits for children with DCD may constrain their ability to perform daily actions that involve dual-tasking. Under the assumption that motor-cognitive integration is compromised in children with DCD, we examined proportional dual-task costs using a novel locomotor-cognitive dual-task paradigm that enlisted augmented reality. We expect proportional dual-task performance costs to be greater for children with DCD compared to their TD peers.

**Methods:**

Participants were 34 children aged 6–12 years (16 TD, 18 DCD) who walked along a straight 12 m path under single- and dual-task conditions, the cognitive task being visual discrimination under simple or complex stimulus conditions presented via augmented reality. Dual-task performance was measured in two ways: first, proportional dual-task costs (pDTC) were computed for cognitive and gait outcomes and, second, within-trial costs (p-WTC) were measured as the difference on gait outcomes between pre- and post-stimulus presentation.

**Results:**

On measures of pDTC, TD children increased their double-limb support time when walking in response to a dual-task, while the children with DCD increased their locomotor velocity. On p-WTC, both groups increased their gait variability (step length and step width) when walking in response to a dual-task, of which the TD group had a larger proportional change than the DCD group. Greater pDTCs on motor rather than cognitive outcomes were consistent across groups and method of dual-task performance measurement.

**Discussion:**

Contrary to predictions, our results failed to support dramatic differences in locomotor-cognitive dual-task performance between children with DCD and TD, with both groups tending to priorities the cognitive over the motor task. Inclusion of a within-trial calculation of dual-task interference revealed an expectancy effect for both groups in relation to an impending visual stimulus. It is recommended that dual-task paradigms in the future continue to use augmented reality to present the cognitive task and consider motor tasks of sufficient complexity to probe the limits of performance in children with DCD.

## Highlights

• Children with DCD demonstrate similar dual-task performance characteristics to TD children.
• Both TD and children with DCD prioritized cognitive over motor tasks.
• The present findings warrant further assessment of augmented-reality dual-task performance in children with DCD during complex motor tasks.

## 1 Introduction

The ability to dual-task (i.e., perform two independent tasks concurrently) is a core aspect of everyday behavior and is critical to safe functioning within our information-driven society. Dual-task performance is underpinned by the development of motor control and cognitive control (i.e., executive functioning) over the course of childhood (Kail, 1991; Anderson, 2002; Adolph and Tamis-LeMonda, 2014; Hagmann-von Arx et al., 2016). Children with Developmental Coordination Disorder (DCD), a chronic and pervasive neurodevelopmental disorder, experience an impaired ability to acquire age-appropriate levels of motor skill (Barnhart et al., 2003). The most commonly reported prevalence of this disorder in school-aged children is at 5–6% (American Psychiatric Association, 2013) and the challenges extend across activities of daily living, participation in sport and leisure pursuits, and classroom tasks that involve motor coordination (Gillberg and Kadesjö, 2003; Zwicker et al., 2013). In children with DCD, there is evidence that both predictive motor control and aspects of cognitive control are not fully developed compared with typically developing (TD) children (Wilson et al., 2013, 2017; Subara-Zukic et al., 2022). Predictive motor control is underpinned by the interplay between brain development and experience (Wolpert et al., 1998; Desmurget and Grafton, 2000; Buneo and Andersen, 2006). When an action is under predictive motor control, a forward estimate (or internal model) of the limb/body position is generated to estimate the expected movement trajectory and its sensory consequences (Shadmehr et al., 2010). Cognitive control involves the ability to control and adapt thoughts, emotions, and behavior, and is synonymous with aspects of executive function, which includes a set of neurocognitive and self-regulatory processes (Hughes and Graham, 2002). As our ability to adjust to dynamic environments and complete novel tasks depends on the integrity of our predictive control mechanisms, these deficits seen for children with DCD may constrain their ability to dual-task. Therefore, cognitive-motor dual-task paradigms can be used to further explore the nature of performance difficulties in children with DCD.

Early work has conceptualized DCD as a disorder of predictive motor control and motor planning; however, the latest reviews challenged the idea that DCD presents as purely a motor disorder (Subara-Zukic et al., 2022; Wilson et al., 2017). The internal modeling deficit (IMD) account (Wilson and McKenzie, 1998; Wilson et al., 2013, 2017) postulates that children with DCD have a core deficit in their ability to implement predictive models of movement, which impairs the automatization of motor skills (Wilson et al., 2013, 2017; Adams et al., 2014). This difficulty may be exacerbated when motor and cognitive tasks are paired, as the literature also reveals deficits in cognitive control in the form of poorer inhibitory control, working memory, and executive attention (Subara-Zukic et al., 2022). Indeed, the integration of cognitive and motor control is problematic for those with DCD (Subara-Zukic et al., 2022) and warrants further investigation, particularly the performance of dual-tasks, which becomes increasingly important with age.

The use of dual-task paradigms in DCD research is relatively under-developed with a small body of published studies, seven reviewed by Schott (2019) and two reviewed by Subara-Zukic et al. (2022). The chosen dual-tasks often incorporate static bipedal motor tasks (Laufer et al., 2008; Tsai et al., 2009; Chen et al., 2012; Chen and Tsai, 2016) or continuous serial calculation or recall cognitive tasks presented through auditory or visual modalities (Laufer et al., 2008; Cherng et al., 2009; Tsai et al., 2009; Chen et al., 2012; Chen and Tsai, 2016; Przysucha et al., 2016; Schott et al., 2016; Kuijpers et al., 2022), with only two studies addressing locomotor-cognitive dual-tasking under complex conditions (Cherng et al., 2009; Krajenbrink et al., 2023). The locomotor-cognitive studies have been limited by a lack of calculation of single-task costs (Cherng et al., 2009) and the use of continuous cognitive tasks that cannot equate a performance cost to a specific phase of the dual-task. In general, higher performance costs under dual-task conditions were evident for children with DCD during complex dual-tasks that challenged postural control (Tsai et al., 2009; Chen et al., 2012; Chen and Tsai, 2016), however, few studies systematically increased task complexity under dual-task conditions. When motor dexterity dual-task complexity was increased, greater performance costs were seen for complex compared to simple task conditions, with the DCD group reporting greater mental effort, but no difference in performance costs was revealed between groups (Krajenbrink et al., 2023). Complex conditions demand more attentional resources and are understood to demonstrate higher dual-task interference effects, particularly for children with DCD (Schott et al., 2016). The DCD dual-task research is, however, limited by a lack of consistent control for single-task differences (Cherng et al., 2009; Tsai et al., 2009; Chen et al., 2012; Chen and Tsai, 2016; Jelsma et al., 2021) and a lack of calculation of proportional dual-task costs (pDTC). The pDTC metric typically involves the comparison of pure single-task performance with dual-task performance (pDTC) and controls for individual or group differences in baseline task performance (Pike et al., 2022). Controlling for single-task performance is important for DCD research to ensure dual-task performance costs do not reflect differences in baseline performance. Taken together, DCD research using dual-task paradigms has been limited by methodological inconsistencies in the choice and classification of tasks, and inconsistent use of the pDTC metric (Schott, 2019).

No study has yet considered a cognitive visual discrimination task presented via augmented-reality under dual-task conditions. This study used a novel locomotor-cognitive dual-task paradigm to examine dual-task performance by demanding predictive motor control and cognitive control. Children were asked to walk down a 12 m walkway while wearing an augmented reality headset, and throughout this period were required to respond verbally to a cognitive stimulus (animal discrimination) that appeared within their visual field. We predicted several hypotheses about the direction and effect of dual-task interference. These predictions were based on previous dual-task research that has consistently shown a larger decline in performance under dual-task conditions for children with DCD compared to TD peers (Cherng et al., 2009; Tsai et al., 2009; Chen et al., 2012; Przysucha et al., 2016; Schott et al., 2016; Kuijpers et al., 2022). The predictions were also informed by deficits in cognitive control (i.e., executive function), seen specifically during inhibitory control, working memory (visual and verbal), and executive attention in individuals with DCD (Rahimi-Golkhandan et al., 2016; Adams et al., 2017; Johnston et al., 2017; Mirabella et al., 2017; Wang et al., 2017). Considering the deficits in predictive motor control in DCD, it was expected that these, combined with cognitive control deficits, would lead to an energy-intensive approach to dual-task performance (Krajenbrink et al., 2023), reducing the ability to share cognitive resources under dual-task conditions (Subara-Zukic et al., 2022). This informed the first and second hypotheses which predicted (1) significant dual-task performance costs on both cognitive and motor outcomes for both TD and DCD groups, and (2) larger dual-task performance costs for DCD compared with TD children. Next, as cognitive tasks are known to be prioritized by children with DCD under dual-task conditions (Laufer et al., 2008; Tsai et al., 2009), the (3) dual-task performance costs were expected to be greater on motor outcomes than cognitive for children with DCD. Furthermore, as research has revealed a greater dual-task performance decline under complex cognitive task conditions for children with DCD (Cherng et al., 2009; Schott et al., 2016), the final hypothesis predicted that (4) larger dual-task performance costs would be evident under complex cognitive task conditions compared with simple, and that children with DCD would be more disadvantaged by complexity than TD children.

## 2 Material and methods

### 2.1 Conceptual framework

A hybrid approach to dual-task performance was adopted to improve the methodological rigor of the dual-task research (Ruffieux et al., 2015; Saxena et al., 2017; Schott, 2019). Informed by the hybrid model of motor skill development and performance (Wilson et al., 2017), we combined cognitive and motor processes under a common conceptual schema and considered the interaction between task (e.g., complexity), environmental (e.g., distractions), and individual (e.g., physical, cognitive) factors. In line with McIsaac et al.’s (2015) dual-task taxonomy, the tasks chosen for this study each had their own unique goal, could be performed in parallel, and differed in novelty and complexity. However, the chosen tasks were understood to recruit from the same “cognitive resource pool” (Wickens, 2008), as they both relied on constant visuo-spatial monitoring and thus, a detriment in performance was predicted to occur. Within the current study, the *environmental* factors were controlled via standardized instructions delivered within a laboratory environment. Factors at an *individual* level, which are implicated in DCD, considered predictive motor control, cognitive control (executive function), and the integration of cognitive-motor control processes. For children with DCD, motor performance relies more heavily on slower, feedback-based control and less on feedforward mechanisms (Wilson et al., 2017). This understanding informed our aforementioned hypotheses and the dual-task paradigm detailed in the following sections. The novelty of this study was the use of a cognitive visual discrimination task presented via augmented reality.

### 2.2 Participants

Children aged 6–12 years (*n* = 34) were recruited via online community advertisements and separated into DCD and TD groups. The DCD group consisted of children who met the research equivalent criteria of DCD (*n* = 18). The research equivalent criteria of DCD was determined via a parent report of motor difficulties, a parent report of no known medical, neurological, or neurodivergent conditions, the child meeting the Movement ABC cut-off criteria, and the brief non-motor assessment of verbal intellectual ability. The TD group consisted of children (*n* = 16) who had no identifiable medical conditions, met the Movement ABC cut-off for typical motor skills, and met the requirements on the brief verbal intellectual ability assessment. An advertisement provided access to a REDCap (Research Electronic Data Capture) (Harris et al., 2009) online screening questionnaire that explained the study, determined eligibility, and obtained informed consent and assent. The advertisement was written to clearly identify participants of interest and the wording was such that pre-diagnosed co-morbid conditions were clearly identified as exclusion factors prior to completing the screening questionnaire. Potential co-morbid medical, physical, and neurodevelopmental diagnoses were controlled for in the participant cohort based on parent reports. Regarding vision, the parents indicated that there were no impairments identified, none of the participants wore glasses, and during familiarization of the cognitive task the participants confirmed that they could see and understand the relevant information. An a-priori power analysis (Faul et al., 2007) revealed that the total sample (*n* = 34) was sufficient based on the desired power of 0.80 and a large (0.80) predicted effect size for differences between TD and DCD groups, as found in previous work (Schott et al., 2016; Jelsma et al., 2021).

### 2.3 Measures

The NICHQ Vanderbilt Assessment Scale-Parent (VADRS-P) was used to assist in determining the risk of Attention Deficit Hyperactivity Disorder (ADHD) in children aged 6–12 years and has demonstrated utility in a pediatric population (Langberg et al., 2010; Kiker, 2011; Limbers et al., 2011; Becker et al., 2012). The 18 ADHD questions were used to screen for inclusion, due to the high co-morbidity of DCD and ADHD (Gillberg, 2003; Martin et al., 2006; Dewey and Bernier, 2016; Smits-Engelsman et al., 2017) and the attentional requirements and specificity of the current study. There were no children excluded based on this questionnaire.

The Weschler Intelligence Scale for Children−Fifth Edition (WISC-V AU&NZ) is a measure of cognitive intelligence, suitable for those aged 6–16 years (Wechsler, 2014). The Verbal Comprehension Index (VCI), which is strongly loaded onto the Full-Scale Intelligence Quotient (FSIQ), was used. The VCI provides a brief, non-motor, general estimate of verbal intellectual ability (Wechsler, 2014). This, in combination with a developmental history, was used to exclude confounding presentations of Intellectual Disability (ID). Thus, those who achieved a VCI ≤ 70 did not meet inclusion for the current study.

The Movement ABC (M-ABC) is a norm-referenced test that identifies children aged 4–12 years with DCD (Henderson and Sugden, 1992). The test uses eight items broken into three sections: (1) manual dexterity, (2) ball skills, and (3) balance. Tasks are differentiated based on four age groups and scaled scores are summed to calculate a total impairment score (TIS). Scores at or above the 16th percentile indicate *No Motor Impairment*, while scores at or less than the 15th percentile indicated *research equivalent DCD*. The MABC’s psychometrics demonstrate strong inter-rater reliability (0.95–1.00) (Smits-Engelsman et al., 2008), sufficient test-retest reliability per age band (0.92–0.98), and “good” concurrent validity (0.60–0.90) (Croce et al., 2001).

The Groton Maze Learning Test (GMLT) is a brief, computer-based test that measures visuospatial processing speed, spatial working memory, and error monitoring in those aged 6–99 years. Performance relies on organizing, integrating, and employing aspects of higher-order complex executive function (Thomas et al., 2016). Participants are required to find a hidden pathway within a 10 x 10 grid of tiles, over five consecutive attempts. Over the five trials, the total error number provides a general measure of executive function, with a lower score denoting stronger performance (Thomas et al., 2016; Wilson et al., 2020). The GMLT was used to provide a measure of executive function for each participant but was not used to define any participant groups. The GMLT has strong utility across the lifespan, has been validated in typically developing (Thomas et al., 2016) and clinical (Schroder et al., 2004) child populations, and is sensitive to age-related changes in executive function (Pietrzak, 2007; Thomas et al., 2013; Wilson et al., 2020; McGuckian et al., 2023).

### 2.4 Locomotor-cognitive dual-task protocol

The Locomotor Single-Task involved walking along a 12 m walkway. The middle 8 m of the walkway featured the GAITRite^®^ system; an instrumented surface comprising pressure-activated sensors that measured the spatiotemporal parameters of gait. Timing of each footstep was measured via activation of the sensors and relative distance between the feet was determined using step pattern data (Webster et al., 2005). A consistent mat length was used, partial steps at the beginning or end of the mat were excluded, and the number of steps taken within the measured period varied between each child according to their natural step length. Participants were required to walk at their preferred speed and completed a minimum of two familiarization trials. If participants appeared to be walking unnaturally, they completed additional familiarization trials until they appeared comfortable. The spatiotemporal analysis of gait was completed in real-time using the paired GAITRite^®^ computer application and data for each step was downloaded and exported to RStudio post-assessment for further analysis. Regular overground walking was chosen due to the high ecological validity of the task, its use across DCD research (Wilmut et al., 2016, 2017; Nieto et al., 2018), and the ability to measure specific characteristics of gait to specify points of cognitive-motor breakdown.

The Cognitive Visual Discrimination (VD) Single-Task was displayed on the HoloLens2*™*, an augmented reality (AR) headset that presents digital visual information overlayed onto real-world surroundings. This method allowed participants to maintain their natural gaze behavior, which is important due to notable differences in fixation and gaze length in the visual control of gait for those with DCD (Subara-Zukic et al., 2022). The novel use of AR within a dual-task paradigm also allowed for improved participant engagement and the ability to capture subtle measures of performance under single- and dual-task conditions (Nenna et al., 2021). The HoloLens2™ was controlled via a custom PsychoPy (Peirce et al., 2019) script initiated via the computer. Once the single cognitive task trial began, the program cycled through eight stimulus presentations to balance motivation and engagement on the Hololens2™, each displayed pseudo-randomly within a 0.4–1.0 s window. A discrete cognitive task was chosen over a continuous task to measure a potential change in performance at a specific point in the dual-task (Abernethy, 1988) and to replicate daily experiences previously used in aging dual-task research (Bock and Beurskens, 2011). The discrete visual discrimination task was developed based on a go/no-go two-choice task that assessed cognitive processing, working memory, and response inhibition (Gomez et al., 2007; Langenecker et al., 2007; Schulz et al., 2007; Raud et al., 2020). Continuous visual discrimination tasks have been successfully used in other developmental dual-task paradigms (Gautier and Droit-Volet, 2002; Fabri et al., 2017; Hallez and Droit-Volet, 2019) and in one DCD dual-task paradigm (Laufer et al., 2008).

**FIGURE 1:**
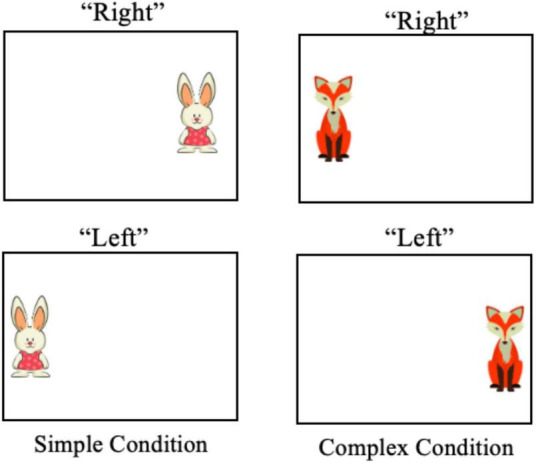
An example of the visual stimuli presented in the simple **(left)** and complex **(right)** conditions.

Children were seated during the cognitive single-task assessment to minimize attentional demands associated with postural control (Reilly et al., 2008). The VD task consisted of both simple and complex conditions. The simple condition (Figure 1; left) required participants to vocally respond (“left” or “right”) via prepotent response to the rabbit when it appeared on the display. That is, participants were required to identify which side of their visual field the rabbit was presented. The complex condition (Figure 1; right) used a response inhibition format. When the fox was displayed, children were required to vocally respond with an opposite (anti) side to where the object was displayed (i.e., “left” when the fox was displayed on the right visual field). The children were instructed to complete this task as quickly and accurately as possible and a storybook was used to introduce the tasks. One block of trials that included four simple and four complex practice trials were completed for familiarization, and participant understanding was confirmed by asking children to repeat the VD task rules to the experimenter. Next, eight video-recorded trials were completed, with four simple and four complex trials presented in a randomized order. After completion of the trials, the Angles (Fulcrum Technologies) video transcoding program was used to review the video recording and calculate response time (ms) and response accuracy (% correct). Cognitive single-task performance for each participant was determined by taking the mean performance for simple and complex tasks, separately.

**FIGURE 2:**
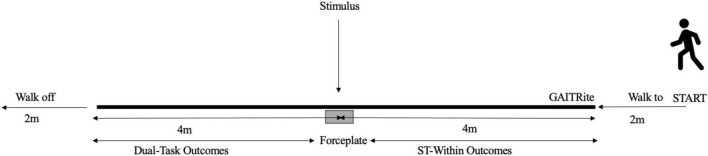
An example of the experimental setup.

The Locomotor-Cognitive VD Dual-Task Trials were completed last. The participants completed eight video-recorded trials (four simple, four complex) where they walked along the GAITRite^®^ mat whilst wearing the HoloLens2™ headset (Figure 2). A single VD stimulus was presented once per walk and was pseudo-randomly presented via the Hololens2™ headset within a 0.4–1.0 s window after the participant stepped onto an inground force plate (AMTI OR6-6, 1000 Hz) situated midway under the GAITRite mat. The participants were instructed to complete the walking and the VD task together were given no advice or recommendation regarding task prioritization. The lead author instructed all participants via the use of a visual storybook that introduced the two tasks, visual discrimination characters and methods of completion to ensure consistency of instructions. Once the story was read to each participant, they were provided with an opportunity to ask questions to ensure they understood the task(s).

The cognitive outcomes included response time (ms) and response accuracy (% correct). Motor outcomes included walking velocity (cm/s), time in double-support (s), stride length (cm), stride time (s), step length variability (cm) and step width variability (cm). Variability metrics were averaged across both legs (left and right). Proportional dual-task costs (pDTC) were calculated for each specific cognitive and motor outcome using the formula: ((dual-task score−single-task score)/single-task score) × 100, with negative values indicating a performance decrement under dual-task conditions. To ensure consistency across the outcomes, the response time, step length and width variability, and double support time outcomes were multiplied by −1 so that negative values always indicated a performance decrement under DT conditions. The pDTC metric was not calculated for the accuracy outcome (% correct) as there were only eight performance values per participant and due to this, the accuracy pDTC values may be misleading. The pDTC metrics were calculated using two different methods to explore the performance patterns−standard and within-trial.

#### 2.4.1 Standard pDTC calculation

The pDTC calculation for the cognitive outcomes was calculated by the mean performance of the seated cognitive task (ST) compared to the mean performance of the cognitive task while walking (DT). For the motor outcome pDTCs, the ST walks (referred to as ST-standard; ST-s) were compared to the steps post-stimulus presentation of DT walks. For the pDTC calculation, the mean ST-s performance was compared to the DT value for each walk and then averaged across each participant’s eight trials before contributing to the group averages.

#### 2.4.2 Within-trial p-WTC calculation

The p-WTC calculation for the motor outcomes was calculated by the difference between the steps before stimulus presentation during the DT walks (referred to as ST-within; ST-w) compared to the steps post-stimulus (DT values) (Figure 2). The p-WTC values were calculated first for each trial, then averaged across each participant’s eight trials and then used to derive the group averages.

### 2.5 Procedure

The testing sessions were completed by the lead author (E.S) who is a registered psychologist. The lead author was first trained by the principal investigator (P.W) in the administration of the MABC and GMLT. The participants completed two testing sessions at Australian Catholic University (ACU), Melbourne Campus. The first involved the baseline measures of the MABC, GMLT, and WISC-V, with a 15-min break offered throughout. One week later, the second session began with leg measurement and a familiarization period with the Hololens2 and GAITRite mat. Next, the single cognitive task was completed (seated), followed by the single motor task (8 walks), and the dual-task paradigm (8 walks), with 15-min breaks provided in between. All children were given a small honorarium upon completion of their involvement as a reimbursement for their time.

### 2.6 Data analysis

RStudio (R Core Team, 2021) was used to analyse the data. Descriptive statistics were calculated to characterize baseline group differences and performance during single- and dual-tasks. The values used were the single- and dual-task performance values and the proportional dual-task costs (pDTC/p-WTC), for both the motor and cognitive tasks. pDTCs are a reliable and valid metric to identify subtle changes in locomotion under dynamic environmental conditions (Pike et al., 2022). The data were examined for missing data points and outliers. Outliers ( ± 3 SDs) were first identified across all participants and removed for single and dual-task results for each outcome variable. The pDTC and p-WTC were next calculated for each motor and cognitive outcome, and outliers were identified and removed (Osborne, 2010).

### 2.7 Statistical analysis

As the data were non-normal, non-parametric Wilcoxon rank-sum tests and linear mixed model (LMM) analyses were used to test the hypotheses. Effect sizes (Wilcoxon *r*) were categorized into small (*r* < 0.10), moderate (*r* = 0.20–0.40) and large effect (*r* > 0.50) categories based on the suggestions of Cohen (1988) and the p-value (*p* < 0.05) was used to determine the statistical significance of the effect. LMMs use a linear regression model that assesses both fixed and random effects, including group effects and participant differences (Peat and Barton, 2008). The proportion of variance explained by the model was categorized as moderate (conditional R^2^ = 0.20–0.40, marginal R^2^ = 0.10–0.25) and substantial (conditional R^2^ > 0.40, marginal R^2^^>^0.25) (Nakagawa and Schielzeth, 2013; Johnson, 2014). This approach was chosen due to its ability to account for repeated measures within participants and handle missing data points (Krueger and Tian, 2004; Vagenas and Totsika, 2018; Bono et al., 2021).

To test hypotheses 1 and 2, the dual-task interference effect on single and dual-task performance was evaluated using non-parametric tests (Wilcoxon *r*), group means, and p-values (*p* < 0.05) for DCD and TD groups. The effect of cognitive task prioritization (Hypothesis 3) and cognitive task complexity (Hypothesis 4) was calculated by comparing the pDTCs between cognitive conditions, and between cognitive and motor outcomes (Wilcoxon *r* and *p*-values). To test whether prioritization differed between groups, the difference between the pDTC of response time and each motor outcome was calculated for each group and compared using Wilcoxon *r*, along with the corresponding effect size (Hypothesis 4). Response time was selected as the primary comparison for the prioritization analysis due to the larger degree of variability observed in the response time pDTCs compared to the accuracy pDTCs, given that response time was a continuous variable and accuracy was limited to an 8-trial average. To test hypothesis 3, single-task, dual-task, and pDTC and p-WTC were compared between DCD and TD groups. This comparison was completed by calculating group means, effect sizes (Wilcoxon *r*), and p-values (*p* < 0.05) using non-parametric tests. Additionally, we employed linear mixed-effects models (LMMs) to fit pDTC and p-WTC metrics, accounting for within-subject correlations between participant trials. The LMM analyses were performed using the *lme*4 package (Bates et al., 2014) in RStudio (R Core Team, 2021). The models aimed to identify the effect of group (DCD or TD) and trial difficulty (simple or complex) on pDTCs for each cognitive and motor outcome variable and incorporated a random intercept for each subject to account for repeated measures across the trials. Covariates of executive function (GMLT Total) and leg length (cm) were also included in the models.

## 3 Results

Table 1 presents descriptive statistics for the TD and DCD groups. There was a significant difference between the groups on MABC Percentile scores, while all other comparisons did not differ significantly.

**TABLE 1:** Descriptive statistics of participant groups.

	TD	DCD	*p*-value
*N*	16	18	
Age M(SD)	8.75 (2.11)	9.44 (1.61)	0.30
**Age Distribution**			
6–7 years	6	2	
8–9 years	4	8	
10–12 years	6	8	
Gender (M/F)	7/9	9/9	0.73
MABC Percentile M(SD)	45.38 (24.37)	6.50 (4.29)	0.00**
Physical activity days/week M(SD)	2.50 (1.09)	2.06 (2.18)	0.45
Physical activity minutes/session M(SD)	63.75 (26.55)	45.00 (47.43)	0.16
Verbal Comprehension Index M(SD)	112.00 (8.47)	110.20 (16.05)	0.69
GMLT Total M(SD)	81.25 (37.97)	81.00 (28.29)	0.98

** Indicates statistical significance at *p* < 0.01.

### 3.1 Single and dual-task performance differences between DCD and TD children

Mean performance outcomes for each group (TD and DCD) under single-task and dual-task conditions, together with pDTC/p-WTC for simple and complex VD tasks, and associated Wilcoxon rank-sum tests, are presented in Tables 2–6.

**TABLE 2:** Cognitive performance outcomes for each group under single-task (ST) and dual-task (DT) conditions, including proportional dual-task costs (pDTCs) and non-parametric Wilcoxon *r* comparisons between groups and conditions.

	Cognitive Task Outcomes						
	**Response Time (ms)**				**Accuracy (% Correct)**		
	**ST** **M (SD)**	**DT** **M (SD)**	**ST v DT** ***(r)***	**pDTC (%)** **M (SD)**	**ST** **M (SD)**	**DT** **M (SD)**	**ST v DT** ***(r)***
**Simple**							
TD	1700 (400)	1550 (280)	0.16	7.85 (10.66)	90.62 (17.97)	96.88 (8.54)	0.18
DCD	1760 (450)	1640 (420)	0.16	6.07 (16.75)	87.50 (19.65)	94.12 (16.61)	0.23
TD v DCD (*r*)	0.08	0.07		0.03	0.09	0.1	
**Complex**							
TD	1770 (400)	1750 (390)	0.04	1.99 (11.42)	92.19 (15.05)	96.88 (8.54)	0.17
DCD	1840 (350)	1700 (500)	0.20	8.27 (23.25)	93.06 (14.36)	95.83 (12.86)	0.14
TD v DCD (*r*)	0.02	0.11		0.28	0.03	0.01	
**Simple v Complex**							
TD (*r*)	0.10	0.31		0.25	0.02	0.00	
DCD (*r*)	0.16	0.04		0.1 8	0.14	0.02	

### 3.2 Cognitive measures

Cognitive task performance, as measured by response time (ms) and accuracy (% correct), did not differ between single and dual-task conditions for the DCD or TD groups (Table 2). Furthermore, under both single and dual-task conditions, performance of the cognitive task was unaffected by stimulus complexity (Table 2).

### 3.3 Spatiotemporal gait measures

Standard single-task (ST-s), dual-task, and pDTC performance for spatiotemporal gait metrics are presented in Table 3. Children with DCD walked significantly faster during DT than ST-s under both complexity conditions. TD children spent significantly more time in double support during DT than ST-s under both complexity conditions. No significant spatiotemporal gait pDTC outcomes were revealed between group or stimulus complexity.

**TABLE 3:** Motor performance outcomes for each group under ST-s and DT conditions, including pDTC and non-parametric comparisons between groups and conditions.

Motor Outcomes																
	**Walk Velocity (cm/s)**				**Double Support (s)**				**Stride Time (s)**				**Stride Length (cm)**			
	**ST-s** **Mean (SD)**	**DT** **Mean (SD)**	**ST v DT** ***(r)***	**pDTC (%)**	**ST-s** **Mean (SD)**	**DT** **Mean (SD)**	**ST v DT** ***(r)***	**pDTC (%)**	**ST-s** **Mean (SD)**	**DT** **Mean (SD)**	**ST v DT** ***(r)***	**pDTC (%)**	**ST-s** **Mean (SD)**	**DT** **Mean (SD)**	**ST v DT** ***(r)***	**pDTC (%)**
**Simple**																
TD	131.71 (12.78)	139.63 (20.04)	0.23	6.37 (14.54)	0.13 (0.03)	0.15 (0.03)	0.40*	−17.49 (12.99)	0.88 (0.09)	0.91 (0.08)	0.13	3.26 (5.89)	115.18 (13.97)	112.11 (17.15)	−0.11	−2.85 (6.56)
DCD	128.52 (10.13)	140.98 (16.54)	0.40*	9.17 (8.99)	0.13 (0.02)	0.15 (0.03)	0.25	−10.48 (12.97)	0.89 (0.08)	0.90 (0.07)	0.10	0.84 (4.78)	114.73 (14.93)	112.69 (14.11)	−0.04	−1.42 (5.50)
TD v DCD (*r*)	−0.14	−0.05		0.16	0.19	−0.02		0.27	0.05	−0.06		−0.20	−0.03	0.07		0.12
**Complex**																
TD	131.71 (12.78)	136.51 (22.05)	0.13	4.08 (14.12)	0.13 (0.02)	0.15 (0.03)	0.41*	−21.80 (16.09)	0.88 (0.09)	0.92 (0.07)	0.20	4.01 (6.35)	115.18 (13.97)	111.31 (17.49)	−0.13	−3.62 (6.35)
DCD	128.52 (10.13)	137.90 (17.33)	0.32*	7.21 (9.46)	0.13 (0.02)	0.15 (0.03)	0.31	−12.43 (9.01)	0.89 (0.08)	0.91 (0.06)	0.17	1.87 (4.88)	114.73 (14.93)	111.48 (15.06)	−0.07	−2.79 (5.28)
TD v DCD (*r*)	−0.14	0.05		0.19	0.19	−0.06		0.27	0.05	−0.09		−0.15	−0.03	0.04		0.08
**Simple v Complex**																
TD (*r*)	0.00	−0.07		−0.04	0.00	0.08		−0.08	0.00	0.07		0.05	0.00	−0.04		−0.05
DCD (*r*)	0.00	−0.11		−0.12	0.00	0.05		−0.10	0.00	0.06		0.11	0.00	−0.03		−0.11

A negative pDTC indicates a performance decrement under dual-task conditions.

* Indicates significance at *p* < 0.05.

For spatiotemporal gait measures within-trial, both the TD and DCD children walked significantly faster after the presentation of the simple cognitive task stimulus compared with pre-stimulus presentation. Children with DCD also demonstrated this effect of faster velocity after the complex cognitive stimulus presentation compared with pre-stimulus velocity (Table 4).

**TABLE 4:** Motor performance outcomes for each group under ST-w and DT conditions, including proportional within-trial comparison (p-WTC) and non-parametric comparisons between groups and conditions.

Motor Outcomes																
	**Walk Velocity (cm/s)**				**Double Support (s)**				**Stride Time (s)**				**Stride Length (cm)**			
	**ST-w** **Mean (SD)**	**DT** **Mean (SD)**	**ST v DT** ***(r)***	**p-WTC (%)**	**ST-w** **Mean (SD)**	**DT** **Mean (SD)**	**ST v DT** ***(r)***	**p-WTC (%)**	**ST-w** **Mean (SD)**	**DT** **Mean (SD)**	**ST v DT** ***(r)***	**p-WTC (%)**	**ST-w** **Mean (SD)**	**DT** **Mean (SD)**	**ST v DT** ***(r)***	**p-WTC (%)**
**Simple (S)**																
TD	124.09 (15.18)	139.63 (20.04)	0.39*	13.24 (7.59)	0.15 (0.03)	0.15 (0.03)	0.04	2.35 (6.56)	0.90 (0.09)	0.91 (0.08)	0.09	0.85 (2.2)	110.97 (15.25)	112.11 (17.15)	0.03	0.94 (3.64)
DCD	125.83 (12.57)	140.98 (16.54)	0.46**	12.02 (8.59)	0.16 (0.03)	0.15 (0.03)	0.11	4.56 (9.36)	0.90 (0.07)	0.90 (0.07)	0.08	0.72 (4.01)	111.73 (14.22)	112.69 (14.11)	0.03	0.30 (3.67)
TD v DCD (*r*)	0.04	0.05		−0.06	0.05	−0.01		0.13	−0.02	−0.05		−0.05	0.07	0.07		−0.08
**Complex (C)**																
TD	123.57 (15.60)	136.51 (22.05)	0.33	10.97 (7.79)	0.16 (0.03)	0.15 (0.03)	0.13	3.93 (8.72)	0.90 (0.07)	0.92 (0.07)	0.14	1.84 (3.6)	110.87 (15.26)	111.31 (17.49)	0.00	0.55 (3.72)
DCD	126.59 (13.53)	137.90 (17.33)	0.36*	9.52 (6.78)	0.16 (0.03)	0.15 (0.03)	0.06	3.29 (7.34)	0.90 (0.07)	0.91 (0.06)	0.06	1.33 (2.77)	112.01 (15.46)	111.48 (15.06)	−0.03	−0.39 (4.2)
TD v DCD (*r*)	0.08	0.05		−0.12	−0.08	−0.06		−0.14	−0.05	−0.09		−0.12	0.07	0.04		−0.17
**Simple v Complex**																
TD (*r)*	0.00	0.07		0.11	0.15	0.08		0.19	0	0.07		0.20	−0.01	−0.04		−0.01
DCD (*r)*	0.04	0.11		0.13	0.03	0.05		0.08	0.02	0.06		0.05	0.02	−0.03		−0.12

A negative pDTC indicates a performance decrement under dual-task conditions.

* Indicates significance at *p* < 0.05 and ** Indicates significance at *p* < 0.01.

### 3.4 Gait variability measures

For the measures of step length variability and step width variability collected during the standard single-task (ST-s) and dual-task performances, there were no differences between the TD and DCD groups for either task complexity condition (Table 5). However, under the simple stimulus condition, participants with DCD exhibited significantly larger pDTCs for both step length variability and step width variability compared with the complex stimulus condition.

**TABLE 5:** Motor performance outcomes for each group under ST-s and DT conditions, including pDTC and non-parametric comparisons between groups and conditions.

	Motor Variability Outcomes							
	**Step Length Variability (cm)**				**Step Width Variability (cm)**			
	**ST-s** **Mean (SD)**	**DT** **Mean (SD)**	**ST v DT** ***(r)***	**pDTC (%)**	**ST-s** **Mean (SD)**	**DT** **Mean (SD)**	**ST v DT** **(r)**	**pDTC (%)**
**Simple (S)**								
TD	2.25 (0.56)	2.75 (0.95)	0.28	−22.58 (33.84)	2.22 (0.52)	2.73 (0.93)	0.29	−22.71 (35.54)
DCD	2.51 (0.56)	2.82 (0.76)	0.19	−12.77 (16.34)	2.48 (0.54)	2.77 (0.70)	0.16	−11.88 (14.64)
TD v DCD (*r*)	0.19		−0.01	0.17	0.21	0.01		0.20
**Complex (C)**								
TD	2.25 (0.56)	2.80 (1.06)	0.25	−21.59 (35.34)	2.22 (0.52)	2.74 (1.02)	0.23	−19.82 (35.43)
DCD	2.51 (0.56)	2.57 (0.65)	0.02	−3.21 (15.73)	2.48 (0.54)	2.57 (0.63)	0.05	−2.54 (13.68)
TD v DCD (*r*)	0.19		−0.09	0.23	0.21	−0.05		0.16
**Simple v Complex**								
TD (*r)*	0.00	0.01		0.02	0.00	−0.01		0.02
DCD (*r)*	0.00	−0.14		0.32*	0.00	−0.15		0.38*

A negative pDTC indicates a performance decrement under dual-task conditions.

* Indicates significance at *p* < 0.05.

For gait variability measures within-trial, both the TD and DCD participants exhibited significantly greater step length variability and step width variability after the presentation of the cognitive task stimulus, compared with the pre-stimulus presentation, under both simple and complex stimulus complexity conditions (Table 6).

**TABLE 6:** Motor performance outcomes for each group under ST-w and DT conditions, including p-WTC and non-parametric comparisons between groups and conditions.

	Motor Variability Outcomes							
	**Step Length Variability (cm)**				**Step Width Variability (cm)**			
	**ST-w** **Mean (SD)**	**DT** **Mean (SD)**	**ST v DT** ***(r)***	**p-WTC (%)**	**ST-w** **Mean (SD)**	**DT** **Mean (SD)**	**ST v DT** ***(r)***	**p-WTC (%)**
**Simple (S)**								
TD	1.63 (0.56)	2.79 (1.01)	0.60**	−108.23 (127.78)	1.53 (0.54)	2.73 (0.93)	0.62**	−297.19 (555.08)
DCD	2.09 (0.72)	2.82 (0.76)	0.49**	−74.47 (72.04)	2.06 (0.77)	2.77 (0.70)	0.50**	−72.94 (70.43)
TD v DCD (*r*)	0.33*	−0.02		0.07	0.36*	0.01		0.42*
**Complex (C)**								
TD	1.75 (0.63)	2.80 (1.06)	0.51**	−87.07 (83.01)	1.67 (0.73)	2.76 (1.07)	0.51**	−175.72 (111.39)
DCD	1.99 (0.63)	2.63 (0.75)	0.38*	−72.31 (48.47)	1.99 (0.63)	2.57 (0.63)	0.42**	−115.72 (149.53)
TD v DCD (*r*)	−0.08	−0.07		0.23	0.23	−0.05		0.36*
**Simple v Complex**								
TD (*r)*	0.17	0.01		0.03	0.07	0.01		0.01
DCD (*r*)	0.06	0.12		0.02	0.00	0.15		0.10

A negative pDTC indicates a performance decrement under dual-task conditions.

* Indicates significance at *p* < 0.05 and ** Indicates significance at *p* < 0.01.

As well, children with DCD demonstrated more variable step lengths and step widths during single-task walking under the simple stimulus condition compared with TD children (Table 6). Analysis of p-WTC metrics showed a moderate, significant group effect on step width variability under both simple and complex stimulus conditions, with TD children demonstrating larger p-WTC deficits than DCD children (Table 6). All other comparisons were non-significant.

### 3.5 Task Prioritization

Results comparing cognitive and motor pDTC are presented in Supplementary Tables 1, 2. The difference between cognitive and motor pDTC values are typically positive, indicating larger pDTC for motor outcomes and therefore a prioritization of cognitive task performance (as measured by response time) for both groups. The smaller pDTC evident for response time (cognitive task) were complemented by significantly larger pDTC for double support time (DCD *r* = −0.51, *p* < 0.01 and TD *r* = −0.75, *p* < 0.01), step length variability (DCD *r* = 0.57, *p* < 0.01 and TD *r* = 0.41, *p* < 0.05), and step width variability (DCD *r* = 0.55, *p* < 0.05 and TD *r* = 0.40, *p* < 0.05) (motor outcomes) for both the TD and DCD groups under simple task conditions. Under complex task conditions, significantly smaller pDTC were reported for both groups for response time compared to double support time (DCD *r* = −0.50, *p* < 0.01 and TD *r* = −0.69, *p* < 0.01), and the DCD group also demonstrated this effect for step width variability (*r* = 0.32 *p* < 0.05). There was no significant difference in prioritization between groups. All other effects were non-significant.

The within-trial p-WTC comparison of both groups revealed a significantly larger effect of step length variability (DCD *r* = 0.64, *p* < 0.01 and TD *r* = 0.79, *p* < 0.01) and step width variability (DCD *r* = 0.61 and, *p* < 0.01 and TD *r* = 0.85, *p* < 0.01) compared to response time under simple stimulus conditions. The same pattern was observed under complex stimulus conditions, with significantly larger effects seen for step length variability (DCD *r* = 0.79, *p* < 0.01 and TD *r* = 0.61, *p* < 0.01) and step width variability (DCD *r* = 0.78 and, *p* < 0.01 and TD *r* = 0.82, *p* < 0.01) compared to response time. For the complex stimulus condition, TD children demonstrated significantly smaller p-WTC on response time compared to walk velocity (*r* = −0.41, *p* < 0.01). However, both cognitive and motor p-WTC metrics were positive indicating a performance improvement under dual-task conditions. All other effects were non-significant.

### 3.6 Linear mixed models

The LMM analyses were conducted to further investigate the hypotheses and model the effect of group, cognitive task difficulty, executive function score, and leg length on pDTCs and p-WTCs. The pDTC of cognitive task response time did not vary as a function of group, cognitive task complexity, or executive function score (Table 7). For pDTC gait metrics (Table 7), leg length was a significant predictor of walking velocity. In contrast, neither cognitive task difficulty nor group predicted walking velocity pDTC metrics, while group, cognitive task difficulty, and leg length were also not significant predictors of double support, step width variability, or step length.

**TABLE 7:** Linear mixed model analyses for pDTCs – between walks.

Fixed Effects	Estimate	Std. Error	df	t	Sig.
**Response Time pDTC**					
Group (TD)	−2.08	4.39	31.24	−0.48	0.64
Cognitive Task Complexity (Simple)	0.43	2.49	217.39	0.17	0.86
Executive Function (GMLT Score)	0.02	0.07	31.96	0.24	0.82
Total explanatory power is moderate (conditional R^2^ = 0.22), and the part related to the fixed effects alone (marginal *R*^2^) is of 0.002. The model’s intercept is at 5.69 [95% CI (−6.96, 18.35)].					
**Walk Velocity pDTC**					
Group (TD)	−2.18	3.33	33.95	−0.65	0.52
Cognitive Task Complexity (Simple)	1.87	1.19	228.39	1.57	0.12
Leg Length	0.67	0.20	34.23	3.32	0.00**
Total explanatory power is substantial (conditional *R*^2^ = 0.55), and the part related to the fixed effects alone (marginal R^2^) is of 0.16. The model’s intercept is at −41.20 [95% CI (−70.25, −12.15)].					
**Double Support pDTC**					
Group (TD)	−7.23	3.92	33.42	−1.89	0.07
Cognitive Task Complexity (Simple)	2.36	1.54	221.16	1.54	0.13
Leg Length	0.26	0.24	33.56	1.09	0.28
Total explanatory power is substantial (conditional *R*^2^ = 0.47), and the part related to the fixed effects alone (marginal R^2^) is of 0.08. The model’s intercept is at −31.54 [95% CI (−65.72, 2.63)].					
**Step Width SD pDTC**					
Group (TD)	−13.57	7.89	33.53	−1.72	0.09
Cognitive Task Complexity Simple)	−5.99	4.17	220.63	−1.44	0.15
Leg Length	0.25	0.48	33.83	0.52	0.60
Total explanatory power is substantial (conditional *R*^2^ = 0.29), and the part related to the fixed effects alone (marginal *R*^2^) is of 0.04. The model’s intercept is at −21.78 [95% CI (−90.78, 47.21)].					
**Step Length SD pDTC**					
Group (TD)	−13.77	7.98	33.39	−1.73	0.09
Cognitive Task Complexity Simple)	−5.22	4.36	221.94	−1.20	0.23
Leg Length	0.23	0.49	33.59	0.48	0.64
Total explanatory power is substantial (conditional *R*^2^ = 0.27), and the part related to the fixed effects alone (marginal R^2^) is of 0.04. The model’s intercept is at −21.73 (95% CI [−91.48, 48.02]).					

** Indicates significance at *p* < 0.01.

For the p-WTC gait metrics (Table 8), cognitive task complexity and leg length were significant predictors of walking velocity, while group was not. In contrast, both group and leg length predicted step width variability p-WTC. Of the examined variables, none were found to be significant predictors of double support or step length variability.

**TABLE 8:** Linear mixed model analyses for p-WTC.

Fixed Effects	Estimate	Std. Error	df	t	Sig.
**Walk Velocity p-WTC**					
Group (TD)	1.69	2.12	33.35	0.80	0.43
Cognitive Task Complexity (Simple)	1.95	0.84	224.92	2.33	0.02*
Leg Length	0.40	0.13	33.75	3.08	0.00**
Total explanatory power is substantial (conditional R^2^ = 0.49), and the part related to the fixed effects alone (marginal R^2^) is of 0.13. The model’s intercept is at −18.66 [95% CI (−37.14, −0.17)].					
**Double Support p-WTC**					
Group (TD)	−0.54	2.28	33.60	−0.24	0.82
Cognitive Task Complexity (Simple)	−0.74	1.28	223.50	−0.58	0.56
Leg Length	0.21	0.14	34.64	1.49	0.15
Total explanatory power is moderate (conditional R^2^ = 0.24), and the part related to the fixed effects alone (marginal R^2^) is of 0.02. The model’s intercept is at −10.69 [95% CI (−30.77, 9.40)].					
**Step Width SD p-WTC**					
Group (TD)	−116.74	46.35	254.00	−2.52	0.01**
Cognitive Task Complexity (Simple)	−3.45	46.16	254.00	−0.08	0.94
Leg Length	−8.70	2.87	254.00	−3.04	0.00**
Total explanatory power is weak (conditional R^2^ = 0.05), and the part related to the fixed effects alone (marginal R^2^) is of 0.05. The model’s intercept is at 531.77 [95% CI (118.10, 945.45)].					
**Step Length SD p-WTC**					
Group (TD)	−28.23	22.50	35.46	−1.25	0.22
Cognitive Task Complexity (Simple)	−11.66	18.22	211.99	−0.64	0.52
Leg Length	−0.23	1.38	36.96	−0.17	0.87
Total explanatory power is weak (conditional *R*^2^ = 0.08), and the part related to the fixed effects alone (marginal R^2^) is of 0.01. The model’s intercept is at −53.09 [95% CI (−252.30, 146.11)].					

* Indicates significance at *p* < 0.05 and ** Indicates significance at *p* < 0.01.

## 4 Discussion

A novel locomotor-cognitive dual-task paradigm was used to explore dual-task interference in children with DCD compared with their TD peers. The cognitive task was presented using augmented-reality technology, a new approach in dual-task and DCD research. Under dual-task conditions, children were required to walk continuously along a straight, flat path and respond to visual stimuli in a discrimination task (presented at the mid-point) using a vocal response. Contrary to our first hypothesis, results showed there was no dual-task interference effect on cognitive outcomes and a varied pattern across motor outcomes (i.e., double support time, walk velocity, stride time, stride length, step width variability, and step length variability) for both DCD and TD groups. Standard evaluation of dual-task interference (pDTC) showed that TD children spent a greater proportion of time in double support while dual-tasking, while children with DCD walked faster when dual-tasking. Within-trial comparison of gait (using p-WTC) showed that both TD and DCD groups walked slower before presentation of the cognitive task compared with after. Children with DCD showed this pattern for both simple and complex cognitive stimuli, whereas for the TD group it was confined to the simple condition only. In contrast to our hypothesis, on measures of step width and step length variability, the TD children showed a larger interference effect (p-WTC) than children with DCD under both simple and complex conditions (Hypotheses 1 and 2). In support of our hypothesis, both groups prioritized the cognitive task more than the motor task under dual-task conditions (Hypothesis 3). Finally, in contrast to our hypothesis there was no effect of cognitive task complexity on cognitive pDTC metrics for either group, contrary to predictions (Hypothesis 4). Discussed below are the effects of task expectancy, specific performance patterns revealed on the pDTC and p-WTC metric, the paradoxical impact of task complexity, and their implications for future dual-task research.

Our findings that children with DCD walked faster under dual-task conditions irrespective of cognitive task complexity and showed *smaller* p-DTCs and p-WTCs on other gait metrics (e.g., stride time, stride length, step length variability, and step width variability) suggests that dual-task effects vary considerably across studies as a function of different task constraints, methodological differences and, possibly, participant sampling. Most notably, our study used a discrete cognitive task, while most others have used continuous tasks in postural (Lajoie et al., 2016) or manual paradigms (Krajenbrink et al., 2023). Earlier studies of postural control report larger performance costs under dual-task conditions for DCD compared with TD groups (Chen et al., 2012; Chen and Tsai, 2016). By comparison, a recent study of manual dexterity found no differences on DTC metrics between TD and DCD groups (Krajenbrink et al., 2023). It is important to note that Krajenbrink et al. (2023), like us, used research criteria to identify children with DCD (i.e., a 16th percentile cut point) whereas Chen and colleagues (Chen et al., 2012; Chen and Tsai, 2016) used the 5th percentile. As well, earlier studies of dual-tasking in DCD often fail to report pDTC outcomes to control for differences in single-task performance (Laufer et al., 2008; Tsai et al., 2009; Chen et al., 2012; Chen and Tsai, 2016; Przysucha et al., 2016; Kuijpers et al., 2022). And, the few studies that have calculated pDTCs vary considerably in their task requirements: static standing while completing a rapid object naming or digit memory task (Chen et al., 2012; Chen and Tsai, 2016); free walking while carrying a tray with/without marbles (Cherng et al., 2009); a Wii Fit game while counting animal sounds or crossing fingers (Jelsma et al., 2021); or completing a Trail-Making-Test or Trail-Walking-Test while connecting numbers or numbers/letters via paper-and-pencil or by cone directed walking (Schott et al., 2016). Finally, in those cases where larger dual-task costs in DCD have been reported, it is on motor outcomes like center-of-pressure sway (Chen et al., 2012; Chen and Tsai, 2016).

### 4.1 Within-trial costs: comparison of gait before and after stimulus presentation

Both groups tended to walk faster after the point of stimulus presentation, and step variability increased (i.e., p-WTC on both step width variability and step length variability). A task expectancy effect may explain this pattern of performance within dual-task trials. In a dual-task context, a task expectancy effect refers to the anticipation of an imminent future event and its influence on movement planning; in effect, the cognitive and motor systems are primed (or prepared) to respond to the presentation of the cognitive task stimulus. We observed that children modified their gait in preparation for the cognitive stimulus by slowing their velocity and maintaining stable step width and length (i.e., reducing variability). This modification may reflect that attention was continuously directed toward anticipating the cue of the cognitive task. As the complexity of the cognitive task was unpredictable, this may also explain the lack of difference in task expectancy between the simple and complex task conditions. Then, once the cognitive task was complete, they reverted to a faster and more natural gait pattern in knowledge that no further stimuli were to be presented for the given trial, a pattern that prioritized speed over gait consistency. Interestingly, under simple task conditions, both groups walked faster, largely by increasing stride length. This increase in velocity (post-cognitive task stimulus) was also associated with a more variable gait pattern (step length and width variability). Taken together, the approach of both groups was to adopt a faster walking speed after stimulus presentation, at the expense of spatial consistency.

Task expectancy, like pre-cue information, provides the performer with predictive information about the upcoming task (whether single or dual), allowing them to make more rapid modifications by reducing the information processing demand at the time of task completion (Abdollahipour et al., 2017; Duma et al., 2020). By expecting a task, the performer is able to plan and prepare, allowing for a more efficient motor movement. Children with DCD have difficulty integrating predictive sensory information (visual, proprioceptive, and vestibular) to maintain balance and produce a consistent gait cycle, which can increase sway, gait variability (Ito et al., 2021; Subara-Zukic et al., 2022), and gait asymmetry (Ito et al., 2023).

Pre-cues can be used, however, to improve motor performance in children with DCD, the effect of which is often assessed by tasks of manual control. For example, Gama et al.’s (2016) research that examined reaction time of a visuomotor task shows that the facilitatory effect of valid pre-cues on motor performance is higher in children with DCD compared with TD. By improving the predictability of task demands, both expectancy and pre-cue information can prime relevant neural circuits and enhance earlier motor planning (Chen et al., 2022). Neural priming is understood to reduce the demand for online motor corrections, a known area of challenge in DCD (IMD hypothesis (Wilson et al., 2013, 2017; Adams et al., 2014)), freeing cognitive resources and allowing for more efficient action. The facilitatory effect of pre-cued motor performance is also supported by high-density EEG data (Duma et al., 2020) that show the brain’s capability to implicitly adjust models of predictive motor control according to the likelihood of an event occurring, which ultimately improves performance of gait and manual dexterity tasks (Gama et al., 2016; Duma et al., 2020). In sum, the results of the current study show that children, regardless of their motor skill status, demonstrate a change in gait velocity in anticipation of having to respond to a cognitive stimulus. Knowledge of the upcoming cognitive stimulus may have acted as a pre-cue that influenced gait patterns before and after its actual presentation. Use of the p-WTC metric in the future may help discern some of the more subtle effects of motor prediction under dual-task conditions.

### 4.2 Gait variability in DCD under dual-task conditions: Does the choice of metric matter?

Group differences on the gait task when performed alone may explain the unexpected pattern we observed for the dual task, relative to previous studies. We showed that children with DCD had a more variable gait pattern under single-task conditions, which is consistent with earlier work showing reduced coordination (Subara-Zukic et al., 2022; Ito et al., 2023) in these children. Increased variability per se may reflect reduced automaticity in the control of gait under lab conditions, an issue exacerbated by poor predictive control of movement (viz IMD hypothesis) (Wilson and McKenzie, 1998; Wilson et al., 2013, 2017). When required to dual-task with the introduction of the cognitive task, step length variability and step width variability increased for the TD group (relative to single-task) to a level comparable to that of the DCD group. Taken together, children with DCD have much less room to increase gait variability without compromising the successful completion of the walking trial itself.

The issue of equating the level of single-task difficulty across groups is prominent in the developmental dual-task literature (Anderson et al., 2011; Saxena et al., 2017). Put simply, if single tasks are titrated for difficulty, any change between single and dual-task performance can be more precisely associated with dual-task ability, rather than group differences in single-task performance. In our study, it was not possible to titrate task difficulty due to the nature and familiarity of locomotion. While, functionally, both groups could complete the task (i.e., get to the end of the walkway), the underlying kinematics were still sensitive to group differences, particularly on spatial variability metrics.

Interestingly, when single-task difficulty was equated between age groups (for each task), age-related changes in dual-tasking were not found consistently (Anderson et al., 2011; Saxena et al., 2017). For example, a dual-task paradigm that paired processing speed and working memory tasks and equated the single-tasks between groups did not reveal an age-related difference between younger (17–27 years) and older (50–81 years) groups for DTCs (Anderson et al., 2011). In the case of manual-cognitive dual-tasking, Krajenbrink et al. (2023) showed that, in the absence of task titration, no DCD-TD group differences were observed on pDTC measures. However, the dual-task performance of children with DCD was seen to require greater mental effort than TD (Krajenbrink et al., 2023). This pattern was similar to that observed in our study on gait variability metrics. In sum, our findings suggest careful consideration of group differences in single-task performance and the need to develop locomotor tasks that can be titrated for difficulty or to use multiple metrics (ST, DT, and pDTC/p-WTC) to better understand the pattern of performance under dual-task conditions. As well, combined results for pDTC and p-WTC suggest that some features of gait variability (i.e., step length variability and step width variability) appear to be more sensitive to dual-task interference than others, a point reinforced in the adult aging literature (Beurskens and Bock, 2013).

### 4.3 The paradoxical effect of task pairing and task complexity

Our lack of group and task complexity effects were unexpected and inconsistent with other work on DCD which has shown a group and complexity effects on motor outcomes (Laufer et al., 2008; Cherng et al., 2009; Tsai et al., 2009; Chen et al., 2012; Schott et al., 2016; Krajenbrink et al., 2023). We consider task pairing and task complexity of both the cognitive and motor task to help explain our findings.

One explanation for the lack of consistent group differences on pDTC relates to the points of measurement during the pairing of a dynamic/continuous motor task with a discrete cognitive task. The pairing of these two tasks was novel in the DCD dual-task research, with previous studies often enlisting continuous cognitive tasks during postural (Chen et al., 2012; Chen and Tsai, 2016; Lajoie et al., 2016) or manual paradigms (Jelsma et al., 2014; Schott et al., 2016; Krajenbrink et al., 2023). No other published dual-task research in DCD had yet combined a dynamic motor with a discrete cognitive task. Evidence suggests that discrete cognitive tasks can measure specific fluctuations in attention and task performance (Abernethy, 1988) and are also relevant to daily experiences, e.g., being distracted by a question or an interruption while walking. Brief distracter cognitive tasks have a demonstrated effect on the dual-task performance of an aging population, with substantial temporal and spatial gait effects post-distracter by seniors compared to young populations (Bock and Beurskens, 2011). Whilst this pattern was also predicted for children with DCD, the assessment of group differences may have been limited by the dynamic and continuous nature of locomotion (Rossignol et al., 2006). A specific fluctuation in attention, if present, may not have been revealed on continuous motor metrics that average the performance of gait velocity, double support time, step width variability, and step length variability. Therefore, dual-task performance deficiencies in DCD may be revealed when a discrete cognitive task is presented at a specific point in a movement cycle, and motor outcomes are measured at this specific point (e.g., during obstacle negotiation).

Another hypothesis to explain the general absence of group differences on pDTC is that the DCD group can complete dynamic dual-tasks under relatively simple task conditions. Simple tasks are defined as tasks that can be achieved automatically, e.g., locomotion. In contrast, a challenging task, such as locomotor obstacle negotiation (Wilmut and Barnett, 2017a,b), enlists greater anticipatory cognitive involvement, internal forward modeling and dynamic visual perception of the object (Patla and Vickers, 2003; Deconinck et al., 2010; da Silva et al., 2011; Higuchi, 2013). Internal forward modeling is a process known to be deficient in children with DCD (Adams et al., 2017; Wilson et al., 2020) and is likely to be reflected by greater performance costs. Similarly, a challenging cognitive task requires efficient cognitive control and may probe complex demands of inhibition, shifting or working memory (Hughes and Graham, 2002), areas of known deficit in DCD (Bernardi et al., 2018; Alesi et al., 2019; Sartori et al., 2020; Fogel et al., 2023), and is likely to demonstrate performance costs, i.e., errors or increased response time. Comparable pDTC between groups suggests that children with DCD can complete relatively simple motor and dual-tasks to the same level as their TD peers, a pattern also seen in motor planning research on DCD (Bhoyroo et al., 2018).

Children with DCD have been shown to perform similarly to their TD peers on simple manual tasks (bar grasping, sword and bar transport) but disadvantaged on more complex single tasks that involve prediction of end-state-comfort (e.g., hexagonal knob task of Krajenbrink et al., 2023 and more complex dual-tasks like completing a pegboard while cycling, for example (Krajenbrink et al., 2023) (see also the review of Bhoyroo et al., 2018). Comparably, the latest consensus review (Subara-Zukic et al., 2022) demonstrated that gait on regular terrain was similar between TD and DCD groups; however, the addition of irregular terrain and targeted foot placements revealed more consistent effects for the DCD group (Gentle et al., 2016; Nieto et al., 2018; Speedtsberg et al., 2018; Parr et al., 2020; Warlop et al., 2020). For example, when walking on uneven terrain, the DCD group were seen to look toward the ground and walk significantly slower with shorter and wider steps compared with their TD peers (Gentle et al., 2016). This approach was discussed as adaptive, with a prioritization of stability and an increase in the extraction of visual information (Gentle et al., 2016). This pattern of performance may be attributed to task complexity and attendant demands on visual attention/sampling. Under dual-task conditions, we would predict that the added cognitive-motor load of complex task constraints (and reduced automaticity of performance) would present a marked challenge for children with DCD. Moreover, as demands on predictive control are increased (for example, by elevating the height of obstacles, or constraining foot placement to specific locations like stepping stones) then we would predict further disadvantage for these children given what we know about their internal modeling issues and apparent immaturity of the motor control system per se (e.g., Wilson et al., 2017; Subara-Zukic et al., 2022).

### 4.4 Limitations

Our final sample of children was moderate from a statistical standpoint, but consistent with other DCD research (e.g., Cherng et al., 2009; Przysucha et al., 2016). Accordingly, effect sizes were generally moderate (Hanel and Mehler, 2019). We also used research criteria to screen children for DCD, which included a range from mild to severe, likely attenuating effect sizes relative to groups classified as severe, only. We acknowledge that the large age range may limit the findings of this study, in particular due to the maturation of motor and cognitive control seen during this developmental period (Wilson and Hyde, 2013; Wilson et al., 2017; Blank et al., 2019); however, the final participant groups were consistent in terms of age range and gender split. We also acknowledge that the small number of trials (at each level of complexity) for the cognitive task may have impacted the stability of performance; however, we considered the importance of participant motivation and energy across an array of trials. Therefore, we are unable to rule out the impact of a potential learning effect of the cognitive task on dual-task performance. Finally, like most studies of dual-tasking in children, we did not randomize the order of single and dual-task presentations. However, we are confident that the tasks were intuitive enough to rule out order effects (see also Krajenbrink et al., 2023).

## 5 Conclusion

The results of the current study highlight that children with DCD and their TD peers both tend to preserve the standard of cognitive task performance under dual-task conditions, while costs are seen on selected gait metrics. TD children spend more time in double support, while children with DCD tended to walk faster when dual-tasking. Within a dual-task trial, both groups modified their gait cycle post-stimulus presentation by walking faster, while step variability increased in both width and length. In general, such estimates of variability appear to be most sensitive to age- and group-related differences in dual-task performance. The results of this research can be used to further inform our clinical understanding of DCD and how children with DCD are able to successfully dual-task similarly to their TD peers when their visual systems are not constrained. Future research may consider dual-task training protocols and vary and extend the level of both cognitive and motor task difficulty in order to pinpoint specific motor control deficits in DCD that compromise dual-task performance. Future research is also recommended to consider gait symmetry variables to explore whether asymmetry is exacerbated for children with DCD (see Wilmut et al., 2017). The continued use of augment-reality to display the cognitive task while measuring a specific point during a locomotor task, for example during obstacle crossing, is recommended to determine if interference is caused by the diversion of attention to the cognitive task. Finally, we encourage continued use of the p-WTC metric to help clarify the use of expectancy information when planning locomotor movements under the complex conditions that define dual-tasking.

## Data availability statement

The raw data supporting the conclusions of this article will be made available by the authors, without undue reservation.

## Ethics statement

The studies involving humans were approved by the Australian Catholic University Human Research Ethics Committee. The studies were conducted in accordance with the local legislation and institutional requirements. Written informed consent for participation in this study was provided by the participants’ legal guardians/next of kin.

## Author contributions

ES-Z: Writing−original draft, Writing−review and editing. TM: Data curation, Formal Analysis, Project administration, Software, Supervision, Writing−original draft, Writing−review and editing. MC: Conceptualization, Data curation, Formal Analysis, Methodology, Supervision, Writing−original draft, Writing−review and editing. BS: Writing−review and editing. PW: Writing−review and editing, Conceptualization, Methodology, Supervision.
