# Supplementary material for: Locomotor-cognitive dual-tasking in children with developmental coordination disorder
Source: Front Psychol. 2024 Mar 6;15:1279427. doi: 10.3389/fpsyg.2024.1279427 (PMC10951910; doi:10.3389/fpsyg.2024.1279427)
Supplement: Supplementary file 1 [file Data_Sheet_1.docx]

**Locomotor-Cognitive Dual-Tasking in Children with Developmental Coordination Disorder**

Supplementary Material

**Table 1.** Task Prioritisation – Comparison between Response Time (Cognitive) pDTC and Motor pDTCs

|  |  | Difference between Response Time pDTC and  Walk Velocity  pDTC  M (SD) | | | *Wilcoxon r* | Difference between Response Time pDTC  and  Double Support  pDTC  M (SD) |  | Difference between Response Time pDTC  and  Step Length Variability  pDTC  M (SD) |  | Difference between Response Time pDTC and  Step Width Variability  pDTC %  M (SD) |  |  |
| --- | --- | --- | --- | --- | --- | --- | --- | --- | --- | --- | --- | --- |
|  |  |  |  |  |  |  | *Wilcoxon r* |  | *Wilcoxon r* |  | *Wilcoxon r* |  |
| **Simple** | |  |  |  | |  |  |  |  |  |  |  |
| TD | | 1.48 (17.76) | | | 0.07 | 25.33 (17.92) | -0.75** | 30.43 (37.31) | 0.41* | 30.56 (38.74) | 0.40* |  |
| DCD | | -3.10 (19.21) | | | -0.11 | 16.55 (21.90) | -0.51** | 18.84 (26.40) | 0.57** | 17.96 (24.84) | 0.55** |  |
| TD v DCD (*r*) | | -0.09 | | |  | -0.18 |  | -0.18 |  | -0.20 |  |  |
| **Complex** | |  |  |  | |  |  |  |  |  |  |  |
| TD | | -2.09 (12.04) | | | -0.07 | 23.80 (14.55) | -0.69** | 23.58 (32.53) | 0.29 | 21.81 (31.65) | 0.23 |  |
| DCD | | 1.06 (20.91) | | | 0.16 | 20.70 (23.59) | -0.50** | 11.48 (26.82) | 0.31 | 10.81 (25.90) | 0.32* |  |
| TD v DCD (*r*) | | 0.20 | | |  | -0.02 |  | -0.16 |  | -0.14 |  |  |

*Note. Values presented within the pDTC M(SD) columns include the difference between the pDTC values. The motor pDTC is consistently deducted from the cognitive pDTC and therefore, a negative prioritisation mean value identifies that the motor pDTC was larger than the response time pDTC.
* Indicates significance at p <0.05 and ** Indicates significance at p <0.01.*

**Table 2**. Task Prioritisation - Comparison between Response Time (Cognitive) pDTC and Motor p-WTCs

|  |  | Difference between Response Time pDTC and  Walk Velocity  p-WTC  M (SD) | | *Wilcoxon r* | | Difference between Response Time pDTC  and Double Support  p-WTC   M (SD) | *Wilcoxon r* | Difference between Response Time pDTC and Step Length Variability  p-WTC   M (SD) | *Wilcoxon r* | Difference between Response Time pDTC and  Step Width Variability  p-WTC %  M (SD) | *Wilcoxon r* |  |
| --- | --- | --- | --- | --- | --- | --- | --- | --- | --- | --- | --- | --- |
|  |  |  |  |  |  |  |  |  |  |  |  |  |
| **Simple** | |  | |  |  | |  |  |  |  |  |  |
| TD | | -5.39 (11.84) | | -0.25 | 5.50 (12.93) | | -0.30 | 116.08 (130.94) | 0.79** | 305.04 (556.31) | 0.85** |  |
| DCD | | -5.95 (14.76) | | -0.25 | 1.51 (17.97) | | -0.12 | 80.54 (82.41) | 0.64** | 79.01 (80.42) | 0.61** |  |
| TD v DCD (*r*) | | -0.05 | |  | -0.08 | |  | -0.09 |  | -0.38* |  |  |
| **Complex** | |  |  |  |  | |  |  |  |  |  |  |
| TD | | -8.97 (10.94) | | -0.41* | -1.94 (11.58) | | 0.09 | 89.06 (84.74) | 0.61** | 117.71 (111.29) | 0.82** |  |
| DCD | | -1.26 (21.63) | | 0.13 | 4.98 (25.18) | | -0.27 | 80.58 (59.73) | 0.79** | 123.99 (151.50) | 0.78** |  |
| TD v DCD (*r*) | | 0.29 | |  | 0.23 | |  | 0.02 |  | -0.34* |  |  |

*Note. Values presented within the pDTC M(SD) columns include the difference between the pDTC values. The motor p-WTC is consistently deducted from the cognitive pDTC and therefore, a negative prioritisation mean value identifies that the motor p-WTC was larger than the response time pDTC.
* Indicates significance at p <0.05 and ** Indicates significance at p <0.01.*
